# Invasive micropapillary carcinoma of the extrahepatic bile duct and its malignant potential

**DOI:** 10.3892/or.2014.3394

**Published:** 2014-08-07

**Authors:** TADASHI YOSHIZAWA, YOSHIKAZU TOYOKI, HIDEAKI HIRAI, TOSHIHIRO HAGA, TAKAHITO TOBA, SHINGO SAKURABA, KENSUKE OKANO, YUNYAN WU, HIROKO SEINO, SATOKO MOROHASHI, KENICHI HAKAMADA, HIROSHI KIJIMA

**Affiliations:** 1 Department of Pathology and Bioscience, Hirosaki University Graduate School of Medicine, Hirosaki 036-8562, Japan; 2 Department of Surgery, Hirosaki University Graduate School of Medicine, Hirosaki 036-8562, Japan

**Keywords:** invasive micropapillary carcinoma, extrahepatic bile duct, lymphatic invasion, lymph node metastasis, prognosis

## Abstract

Invasive micropapillary carcinoma (IMPC) was originally described as a distinctive type of invasive carcinoma in the breast, but it has not been recognized as a histological type of the extrahepatic bile duct cancer. The present study demonstrated clinicopathological features and patient prognosis of IMPC. We examined histological reviews of 93 consecutive cases of the extrahepatic bile duct cancer and identified 13 cases which included IMPC component. The component of IMPC ranged from 5 to 60% of the primary tumor tissue, which was mainly detected at the invasive front of the tumor. Of the 13 cases, 12 (92.3%) carcinomas with IMPC showed lymph node metastasis more frequently compared to conventional adenocarcinoma (39.2%, P<0.001). Presence of IMPC component was significantly associated with poor overall survival (P=0.003). In conclusion, extrahepatic bile duct carcinoma with IMPC component showed significant lymphatic invasion, lymph node metastasis, and resulted in poor prognosis.

## Introduction

Extrahepatic bile duct carcinoma is an epithelial cancer originating from the bile ducts with features of cholangiocytic differentiation. There is no significant geographical variation in the incidence of extrahepatic bile duct carcinoma. In the USA, extrahepatic bile duct carcinoma accounts for 0.16% of all invasive cancers in males and 0.15% in females in the general population (1). Surgical treatment is the only curative therapy for extrahepatic bile duct carcinoma and is therefore the treatment of choice if feasible. The spreading cancer cells via the lymphatic to regional lymph nodes are an important factor for tumor progression. In a recent study, the median disease-specific survival rate after surgery in patients with lymph node metastasis was lower than that of patients without lymph node metastasis (19.3 vs. 53.5 months, P<0.0001) (2).

Recent studies have demonstrated that an invasive micropapillary carcinoma (IMPC) frequently shows aggressive tumor behaviors with marked lymph-vascular invasion, resulting in poor prognosis in several organs including the breast (3–5), urinary bladder (6–8), lung (9–13), parotid gland (14,15), pancreas (16), gallbladder (17), colorectum (18) and stomach (19–22). To the best of our knowledge, however, there has been only one case report of extrahepatic bile duct carcinoma which contains IMPC (23). Therefore, the clinicopathological significance of IMPC has not yet been elucidated in extrahepatic bile duct. To clarify the significance of IMPC in extrahepatic bile duct, the present study investigated the clinicopathological features of 13 cases of extrahepatic bile duct carcinoma containing IMPC, compared with 80 cases of conventional extrahepatic bile duct carcinoma.

## Materials and methods

### Patients

We investigated consecutive bile duct carcinoma surgical cases treated between January 2007 and December 2012, after obtaining each patient’s informed consent for use of their clinical records and pathological specimens at Hirosaki University Hospital. The series consisted of 69 men and 24 women with a median age of 70 years (range, 31–83 years). The carcinomas were located in the perihilar (34 cases) and distal bile duct (59 cases), according to the anatomic location (24). Curative resection and regional lymph node dissection were dependent on the location of primary tumors: pancreaticoduodenectomy or pylorus-preserving pancreaticoduodenectomy was performed in 56 patients, bile duct resection in 1 patient and combined hepatectomy with bile duct resection in 29 cases and combined hepatectomy and pancreaticoduodenectomy in 7 patients. Survival data were obtained from hospital medical charts and the median observation period was 25.6 months (79 cases).

### Pathological analysis

All surgically resected specimens were routinely fixed with 10% formalin, then embedded in paraffin and stained with hematoxylin and eosin (H&E) for pathological evaluation. The following histological features were assessed: depth of invasion (T-grade), histological type, lymphovascular invasion, perineural invasion, the mode of infiltration pattern, lymph nodal metastasis and IMPC component. We defined IMPC as tumor clusters of tumor cells lying within clear spaces. The clear spaces have no endothelial lining. Cases with tumor clusters only in the lymphovascular channels or mucinous lesions, which resemble the IMPC pattern, were excluded. We also evaluated the component ratio of IMPC in the entire tumor tissues. In cases where the distinction of lymphovascular invasion from the IMPC was difficult based on H&E stained alone, we evaluated the cases with immunohistochemical podoplanin (D2-40) staining. Degrees of lymphatic, vessel and perineural invasion were classified as: 0, no invasion; 1, mild invasion; 2, moderate invasion; 3, severe invasion. Modes of infiltration pattern were classified into three groups, i.e., INF-α, cancer nests showing expansive growth and presenting the clear borderline between the tumor tissue and stroma; INF-β, intermediate patterns of growth and invasive structure of INF-α and INF-γ; and INF-γ, scirrhous growth with unclear borderline of the invasive front. These data were evaluated according to our previous study (25) and the General Rules for Surgical and Pathological Studies on Cancer of the Biliary Tract (26) with reference to the World Health Organization classification and staged according to the TMN classification of the International Union Against Cancer (UICC) (24). We also investigated phenotypes of IMPC components using the immunohistochemical procedure, as described below.

### Immunohistochemistry

For histological examination, extrahepatic bile duct carcinoma specimens were routinely fixed with formalin, embedded in paraffin and thin-sectioned. Sections 4-μm-thick were mounted on saline-coated glass slides. Immunohistochemical examination was performed on deparaffinized sections using the standard avidin-biotin-peroxidase complex method with automated immunostainer (Benchmark XT; Ventana Medical System, Tucson, AZ, USA). The characteristic ‘inside-out’ pattern of IMPC was confirmed with the immunohistochemical MUC1 antibody. Furthermore, we investigated the phenotypes of IMPC using MUC1, MUC2, MUC5AC and MUC6 antibodies. Podoplanin (D2-40) was used for clarifying lymphatic invasion. The antibodies we used were: MUC1 (1:50, clone Ma696), MUC2 (1:50, clone Ccp), MUC5AC (1:100, clone CLH2), MUC6 (1:100, clone CLH5; all from Novocastra Laboratories), D2-40 (1:100, clone D2-40; Dako, Glostrup, Denmark).

### Evaluation of immunohistochemistry

Luminal membranous immunoreactivities of the tumor were judged as positive for MUC1, and cytoplasmic immunoreactivities as positive for MUC2, MUC5AC and MUC6. The results were classified into two groups based on the percentage of positive cells with each staining as follows: negative group in which <5% of cancer cells were stained, and positive group in which ≥5% was stained.

### Statistical analysis

Statistical comparisons between two groups were analyzed using the Pearson’s Chi-square test for categorical data and the Mann-Whitney test for continuous data. Survival curves were constructed using the Kaplan-Meier method and differences in survival were evaluated using the log-rank test. The relative prognostic factors were analyzed with the Cox proportional hazards regression model. Differences were considered to be statistically significant if the P-value was <0.05. All statistical evaluations were performed using R (http://www.r-project.org) and PASW statistics software (version 18.0; SPSS, Inc., Chicago, IL, USA).

## Results

### Histological and immunohistochemical findings of IMPC

**Figure 1:**
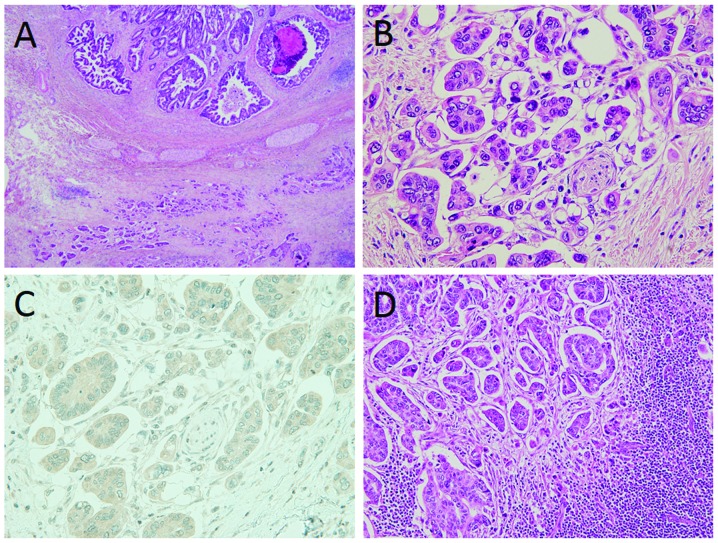
Histological findings of IMPC of the extrahepatic bile duct. IMPC component was found at the invasive front of the tumor (A, top left). Cancer cells were characterized by small round to ovoid micropapillary tumor cell clusters with no fibrovascular cores, lying within clear stromal spaces (B, top right). Immunohistochemical staining for D2-40 revealed the absence of endothelial lining cells in clear spaces surrounding the tumor clusters (C, bottom left). Lymph node metastasis also consisted of the IMPC (D, bottom right). The tumor clusters were similar to the primary lesion. IMPC, invasive micropapillary carcinoma.

**Figure 2:**
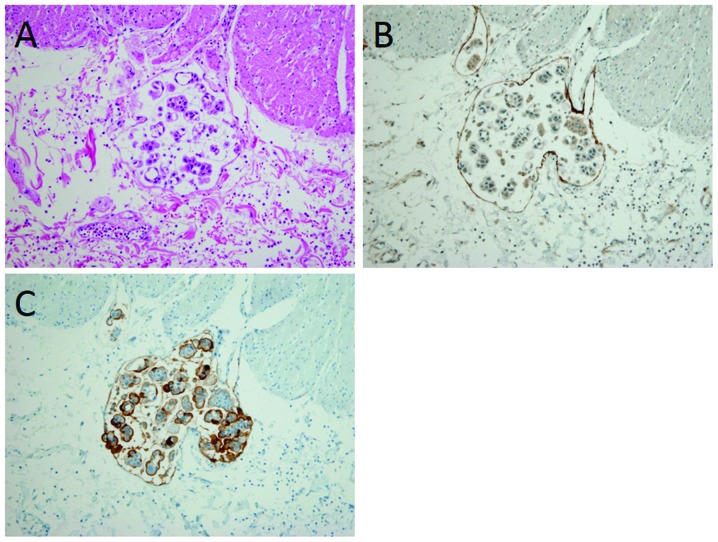
Marked lymphatic invasion of IMPC component was detected (A, top left), immunohistochemical staining for D2-40 revealed the endothelial lining cells (B, top right). Immunohistochemical staining for MUC1 revealed the ‘inside-out’ pattern (C, bottom left). IMPC, invasive micropapillary carcinoma.

We reviewed 93 cases of the extrahepatic bile duct cancer and found 13 cases (14.0%) with IMPC component. The clinicopathological findings are summarized in Table I. The 13 patients (11 men and two women) with IMPC ranged in age from 49 to 80 years (mean, 68 years). Follow-up information was available for 10 patients, with a median follow-up time of 17 months (range, 2–54 months). Eight patients succumbed to the disease, one was alive with disease and one was alive without recurrent disease. Histologically, IMPC components ranging from 5 to 60% (mean ± SD, 17.7±15.4, data not shown) of the entire tumor were mainly found at the front of the tumor (Fig. 1A). The tumor was characterized by small round to ovoid micropapillary tumor cell clusters with no fibrovascular cores, lying within clear stromal spaces (Fig. 1B). The clear stromal spaces resembled lymphatic vessels, but were immunohistochemically negative for D2-40, a marker of lymphatic vessel (Fig. 1C). Metastatic carcinomas of lymph nodes also had IMPC component (Fig. 1D). The carcinoma cells characteristically displayed a reverse polarity, known as an ‘inside-out’ growth pattern, mimicking extensive lymphatic invasion (Fig. 2A and B).

**Figure 3:**
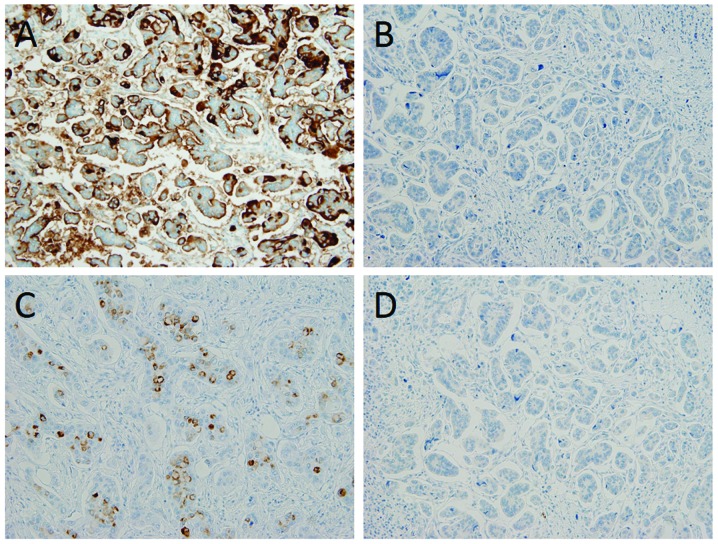
Immunohistochemical findings of a representative case. The ‘inside-out’ structures of IMPC were positive for MUC1 at peripheral cell membranes (A, top left), negative for MUC2 (B, top right), focally positive for MUC5AC (C, bottom left) and negative for MUC6 (D, bottom right). IMPC, invasive micropapillary carcinoma.

The results of immunohistochemistry are summarized in Table II. Nine (69.2%) of the 13 cases of IMPC were positive for MUC1. MUC1 immunoreactivity was predominantly detected at the surface of the cell cluster and clearly exhibited the ‘inside-out’ growth pattern (Figs. 2C and 3A). MUC5AC was focally found in the cytoplasm, as well as at the cell surface, in 4 of the 13 cases (Fig. 3C). MUC2 and MUC6 staining was negative in all cases of IMPC (Fig. 3B and D).

### Clinicopathological findings of IMPC

The clinicopathological findings of extrahepatic bile duct carcinoma with and without IMPC component are summarized in Table III. The presence of IMPC component was significantly correlated with lymph node metastasis, lymphatic invasion and the mode of infiltration pattern (P<0.001, P=0.016 and P=0.027, respectively). In addition, the extrahepatic bile duct cancer with IMPC component frequently showed lymph node metastasis with IMPC component (Table IV, P<0.001).

### Univariate and multivariate analysis

**Figure 4:**
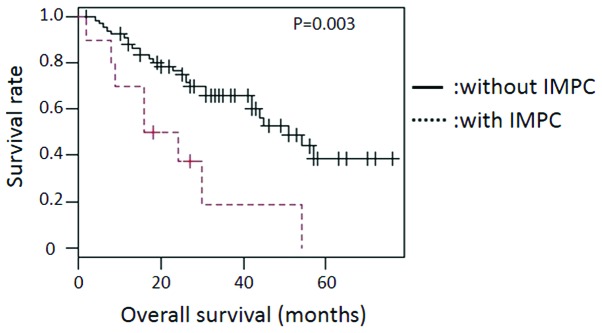
OS rate of patients with IMPC component. Patients with IMPC component showed reduced OS (P=0.003). OS, overall survival; IMPC, invasive micropapillary carcinoma.

**Figure 5:**
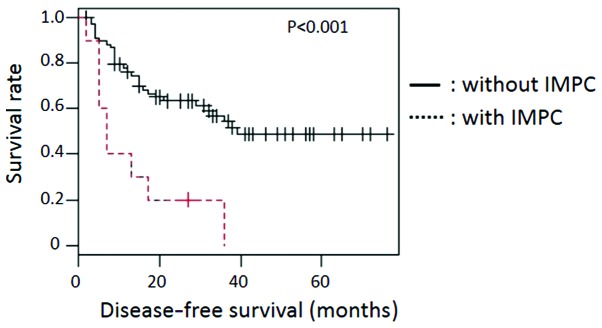
DFS rate of patients with IMPC component. Patients with IMPC component showed reduced DFS (P<0.001). DFS, disease-free survival; IMPC, invasive micropapillary carcinoma.

Survival curves based on univariate survival analysis demonstrated that the patients with IMPC were associated with poor prognosis (Fig. 4, P=0.003) and poor disease-free survival (Fig. 5, P<0.001). To clarify potential prognostic indicators, we analyzed various pathological factors investigated (Table V). Univariate analysis revealed that the following factors were correlated with poor prognosis: IMPC component [relative risk (RR) 3.195, 95% confidence interval (CI) 1.437–7.107, P=0.004], depth of invasion (RR 3.261, 95% CI 1.583–6.719, P=0.001), histological type (RR 6.787, 95% CI 2.072–22.23, P=0.002), lymphatic invasion (RR 4.028, 95% CI 1.973–8.224, P<0.001), venous invasion (RR 2.714, 95% CI 1.327–5.551, P=0.006) and lymph node metastasis (RR 3.868, 95% CI 1.946–7.684, P<0.001).

## Discussion

In the present study, we clarified clinicopathological characteristics of IMPC of the extrahepatic bile duct. We clarified that IMPC frequently showed aggressive tumor growth with lymphatic invasion and lymph node metastasis, resulting in short overall/disease-free survival of the patients. This is the first report describing clinicopathological malignant potential of IMPC of the extrahepatic bile duct.

IMPC component has been reported in several organs, such as breast, urinary bladder, lung, parotid gland, pancreas, gallbladder, colorectum and stomach (3–22). The previous reports revealed that IMPC exhibited a tendency for lymphatic invasion and lymph node metastasis. In our study, IMPC component of extrahepatic bile duct ranged from 5 to 60% of the entire tumor, while with IMPC components were found in the other organs: 10–90% in salivary duct carcinoma (15), 10–90% in gastric carcinoma (21), 5–10% in gallbladder carcinoma (17), 5–95% in breast cancer (27). IMPC component, regardless of its tumor volume, has been shown to have malignant potential for lymphatic invasion and lymph node metastasis.

The molecular mechanisms of the malignant potential of IMPC have not yet been fully elucidated, while IMPC is histologically characterized by the ‘inside-out’ pattern. A previous study proposed that extracellular matrix (ECM) contributed to the IMPC structure (28). Madin-Darby canine kidney (MDCK) cells exhibited ‘inside-out’ structure without type I collagen, but were able to reorient their cell polarity under the presence of type I collagen in ECM. Here, the reorientation of cell polarity was shown to be related to RAC1, PI3-kinase and a PKC (28). Cancer cells of IMPC may have abnormalities of RAC1 suppression cascade and show the characteristic ‘inside-out’ structures (29). We also revealed that MUC1 expression was predominantly at the surface of tumor clusters. The MUC1 expression was similar to the other organs (30). Human MUC1 is a high molecular weight transmembrane glycoprotein, which is apically expressed in the majority of glandular epithelia (31). Increased MUC1 expression has been shown to inhibit integrin-mediated cell adhesion between cancer cells and ECM (32) and to decrease adhesion to type I collagen (33). Based on this evidence, IMPC expressing MUC1 may reduce the cell adhesion from ECM and result in forming the characteristic ‘inside-out’ structures.

Our study revealed that IMPC structures were found not only in the primary carcinoma lesions, but also in the foci of lymphatic vessels and metastatic lymph nodes. The ‘inside-out’ IMPC structures were thought to play an important role in the lymph node metastasis. Of note, IMPC exhibited stromal desmoplastic reactions around the ‘inside-out’ cancer cell clusters. The desmoplastic changes consisted of proliferation of fibroblasts and collagen fibers and were found not only in the primary lesion, but also in the parts of lymph node metastasis. The desmoplastic changes are thought to be associated with epithelial-mesenchymal transition, which may contribute to an aggressive growth of the invasive cancer.

The results of univariate analysis revealed that IMPC was significantly correlated with poor patient prognosis, but the multivariate analysis using Cox proportional hazards model showed that IMPC was not an independent prognostic factor for overall survival. We suspected the reason why it was not an independent factor was that IMPC may be strongly associated with lymphatic invasion and lymph node metastasis. An important clinical issue is that the presence of IMPC indicates malignant potential, even if the component is small. Therefore, pathologists should describe presence of IMPC component in the diagnostic report, even if the component is a small part of the extrahepatic bile duct carcinoma.

## Figures and Tables

**Table I:** Extrahepatic bile duct cancer with IMPC component (13 cases).

Case No.	Age/gender	Percentage of IMPC component	Lymph node metastasis	Survival (months)	Status
1	74/M	50–60	9/17	2	Deceased
2	49/F	40–50	6/29	54	Deceased
3	71/M	20–30	2/6	N/A	N/A
4	70/M	10–20	2/6	24	Deceased
5	65/M	10–20	1/8	7	Deceased
6	70/M	10–20	1/10	18	Alive
7	72/M	10–20	3/23	N/A	N/A
8	72/F	5–10	2/21	30	Deceased
9	73/M	5–10	3/39	8	Deceased
10	73/M	5–10	0/19	27	Alive
11	43/M	5–10	1/10	16	Deceased
12	74/M	5–10	4/34	9	Deceased
13	80/M	5–10	1/14	N/A	N/A

IMPC, invasive micropapillary carcinoma; M, male; F, female; N/A, not applicable.

**Table II:** Immunohistochemical characteristics of IMPC component of extrahepatic bile duct cancer (13 cases).

Case No.	Age/ gender	Mucin phenotype			

		MUC1	MUC2	MUC5AC	MUC6
1	74/M	+	−	+	−
2	49/F	−	−	−	−
3	71/M	−	−	−	−
4	70/M	+	−	−	−
5	65/M	+	−	+	−
6	70/M	+	−	−	−
7	72/M	+	−	−	−
8	72/F	+	−	+	−
9	73/M	−	−	−	−
10	73/M	+	−	−	−
11	43/M	−	−	−	−
12	74/M	+	−	+	−
13	80/M	+	−	−	−

IMPC, invasive micropapillary carcinoma; M, male; F, female; Mucin phenotypes: MUC1, biliary; MUC2, intestinal; MUC5AC, gastric foveolar; MUC6, gastric pyloric.

**Table III:** Histopathological characteristics of extrahepatic bile duct cancer with or without IMPC component.

Variables	With IMPC component (n=13)	Without IMPC component (n=80)	P-value
Age			0.956
>65	3	58	
≤65	10	22	
Gender			0.503
Male	11	58	
Female	2	22	
Location			0.361
Perihilar^a^	3	31	
Distal^a^	10	49	
Depth of invasion			0.071
T1 or T2^b^	3	40	
T3 or T4^b^	10	40	
Histological type			0.332
Well-differentiated adenocarcinoma	2	27	
Other histological type	11	53	
Lymph node metastasis			<0.001
pN (+)	12	31	
Lymphatic invasion			0.016
ly0 or ly1	2	41	
ly2 or ly3	11	39	
Venous invasion			0.063
v0 or v1	2	34	
v2 or v3	11	46	
Perineural invasion			0.332
n0 or n1	2	27	
n2 or n3	11	53	
INF^c^			0.027
α or β	1	34	
γ	12	46	

IMPC, invasive micropapillary carcinoma;

a according to WHO classification;

b according to TNM classification;

c mode of infiltration pattern, as described in Materials and methods.

**Table IV:** Histological relationship between primary tumor and lymph node metastasis with or without IMPC component.

	With IMPC component (n=13)	Without IMPC component (n=80)	P-value
Lymph node status			
pN (+)	12 (92.3%)	31 (38.8%)	<0.001

	Primary with IMPC component (%)	Primary without IMPC component (%)	P-value

Lymph node with IMPC component	11 (25.6)	1 (2.3)	<0.001
Lymph node without IMPC component	1 (2.3)	30 (69.8)	

IMPC, invasive micropapillary carcinoma.

**Table V:** Univariate and multivariate analysis of prognostic factors of survival.

Variables	Values (%)	Univariate analysis P-value	Multivariate analysis P-value
IMPC component		0.004	0.643
With IMPC component	10 (12.7)		
Without IMPC component	69 (87.3)		
Depth of invasion		0.001	0.234
T1 or T2^a^	40 (50.6)		
T3 or T4^a^	39(49.6)		
Histological type		0.002	0.062
Well-differentiated adenocarcinoma	24 (30.1)		
Other histological type	55 (69.6)		
Lymphatic invasion		<0.001	0.223
ly0 or ly1	43 (54.4)		
ly2 or ly3	36 (45.6)		
Venous invasion		0.006	0.76
v0 or v1	36 (45.6)		
v2 or v3	43 (54.4)		
Perineural invasion		0.071	-
n0 or n1	29 (36.7)		
n2 or n3	50 (63.3)		
INF^b^		0.064	-
α or β	30 (38.0)		
γ	49 (62.0)		
Lymph node metastasis		<0.001	0.116
pN (−)	50 (63.3)		
pN (+)	29 (36.7)		

IMPC, invasive micropapillary carcinoma;

a according to TNM classification;

b mode of infiltration pattern, as described in Materials and methods.
